# TGF‐β gene transfer and overexpression *via* rAAV vectors stimulates chondrogenic events in human bone marrow aspirates

**DOI:** 10.1111/jcmm.12774

**Published:** 2016-01-25

**Authors:** Janina Frisch, Ana Rey‐Rico, Jagadeesh Kumar Venkatesan, Gertrud Schmitt, Henning Madry, Magali Cucchiarini

**Affiliations:** ^1^ Center of Experimental OrthopaedicsSaarland University Medical CenterHomburg/SaarGermany; ^2^ Department of Orthopaedic SurgerySaarland University Medical CenterHomburg/SaarGermany

**Keywords:** cartilage repair, gene therapy, bone marrow aspirates, rAAV, TGF‐β

## Abstract

Genetic modification of marrow concentrates may provide convenient approaches to enhance the chondrogenic differentiation processes and improve the repair capacities in sites of cartilage defects following administration in the lesions. Here, we provided clinically adapted recombinant adeno‐associated virus (rAAV) vectors to human bone marrow aspirates to promote the expression of the potent transforming growth factor beta (TGF‐β) as a means to regulate the biological and chondrogenic activities in the samples *in vitro*. Successful TGF‐β gene transfer and expression *via* rAAV was reached relative to control (*lacZ*) treatment (from 511.1 to 16.1 pg rhTGF‐β/mg total proteins after 21 days), allowing to durably enhance the levels of cell proliferation, matrix synthesis, and chondrogenic differentiation. Strikingly, in the conditions applied here, application of the candidate TGF‐β vector was also capable of reducing the hypertrophic and osteogenic differentiation processes in the aspirates, showing the potential benefits of using this particular vector to directly modify marrow concentrates to generate single‐step, effective approaches that aim at improving articular cartilage repair *in vivo*.

## Introduction

The adult articular cartilage has a limited ability for self‐repair 1 as a result of the absence of vascularization in this highly specialized tissue. Options to provide reparative cells in sites of cartilage lesions include autologous chondrocyte implantation and marrow‐stimulating techniques to recruit chondroregenerative bone marrow‐derived mesenchymal stem cells (MSCs) 2, 3, 4 but the outcome of such procedures do not meet the original hyaline cartilage as the repair tissue is commonly made of a fibrocartilaginous structure (type‐I collagen instead of type‐II collagen proteoglycans) of lesser mechanical quality and that does not integrate with the adjacent, original cartilage 5. A promising approach might be to conveniently supply the lesions with bone marrow concentrates 6 composed of chondrogenically competent MSCs (~1%) that specifically commit towards the chondrocyte phenotype under adapted treatment conditions among other cell populations (haematopoietic progenitor cells, haematopoietic cells, fibroblasts) 7, 8 rather than using isolated progenitors that necessitate more complex steps of preparation and expansion 6. Yet, despite encouraging attempts to treat patients with bone marrow concentrates 9, 10, 11, 12, 13, 14, 15, restoration of the articular cartilage tissue in its full integrity has not been reported to date, showing the urgent need for novel, effective regimens.

Direct genetic modification of bone marrow aspirates to overexpress chondrogenic and/or chondroreparative factors may offer improved tools to enhance the healing response in sites of cartilage injury upon local transplantation 16, 17, 18. In marked contrast with the substantial information available on gene transfer to target isolated MSCs, little is known on the possibility to transduce marrow concentrates as a less invasive approach for improved cartilage repair. While various groups reported the ability of adenoviral vectors to mediate gene transfer in rabbit and sheep models 19, 20, 21, showing effective but relatively short‐term transgene (transforming growth factor beta – TGF‐β, bone morphogenetic protein 2 – BMP‐2, Indian hedgehog – IHH) expression levels (some days) at high (10^10^–10^11^) vector doses, we recently tested the potential of applying recombinant vectors derived from the human non‐pathogenic adeno‐associated virus (AAV) to human marrow aspirates in the light of the reduced toxicity and immunogenicity of this vector class that can be maintained over extended periods of time as stable and safe episomes 18. Specifically, we showed that rAAV can achieve elevated (~90%) gene transfer efficiencies and durable (125 days) transgene expression at much lower (8 × 10^5^) vector doses in the absence of adverse reactions 22, allowing for enhanced chondrogenic differentiation upon therapeutic gene delivery of the transcription factor SOX9 22 of and insulin‐like growth factor I (IGF‐I) 23.

In this study, we tested the ability of the potent chondrogenic and chondroreparative TGF‐β factor 24, 25, 26, 27 to stimulate the biological and chondrogenic activities in human marrow aspirates *via* rAAV gene transfer in the light of our previous findings showing activation of such processes when applying the same vector to isolated hMSCs 28. Our results show that administration of rAAV promotes an effective, significant production of TGF‐β, leading to enhanced levels of proliferation, biosynthesis and chondrogenic differentiation in the aspirates while reducing hypertrophy/terminal differentiation and osteo‐/adipogenic events, showing the value of applying such samples in site of cartilage damage during transplantation protocols.

## Materials and methods

### Reagents

All reagents were from Sigma‐Aldrich (Munich, Germany), unless otherwise identified. Recombinant TGF‐β (rTGF‐β) was purchased at Peprotech (Hamburg, Germany) and the dimethylmethylene blue dye at Serva (Heidelberg, Germany). The anti‐TGF‐β (V) and anti‐SOX9 (C‐20) antibodies were from Santa Cruz Biotechnology (Heidelberg, Germany), the anti‐type‐II collagen (II‐II6B3) antibody from the NIH Hybridoma Bank (University of Iowa, Ames, USA), the anti‐type‐I collagen (AF‐5610) antibody from Acris (Hiddenhausen, Germany) and the anti‐type‐X collagen (COL‐10) from Sigma‐Aldrich. Biotinylated secondary antibodies and the ABC reagent were obtained from Vector Laboratories (Alexis Deutschland GmbH, Grünberg, Germany). The hTGF‐β Quantikine ELISA was from R&D Systems (Wiesbaden, Germany) and the type‐II, ‐I and ‐X collagen ELISAs from from Antibodies‐Online (Aachen, Germany).

### Plasmids and rAAV vectors

All plasmids are based on the same parental AAV‐2 genomic clone, pSSV9 29, 30. rAAV‐*lacZ* carries the *lacZ* gene encoding the *Escherichia coli* β‐galactosidase (β‐gal) and rAAV‐hTGF‐β a human TGF beta 1 (hTGF‐β) cDNA fragment (1.2 kb), both under the control of the cytomegalovirus immediate‐early (CMV‐IE) promoter 22, 23, 28, 31. Conventional (not self‐complementary) rAAV vectors were packaged using the 293 adenovirus‐transformed embryonic kidney cell line. Helper functions were provided by Adenovirus 5 in combination with rep and cap functions of a pAd8 helper plasmid as previously described 28. Purification, dialysis and titration of the vector preparations *via* real‐time PCR were performed, averaging 10^10^ transgene copies/ml with approximately 1/500 functional recombinant viral particles 22, 23, 28, 31.

### rAAV‐mediated gene transfer

Bone marrow was aspirated from the distal femurs of patients undergoing total knee arthroplasty (~10 ml, *n* = 3). All patients included in the study provided informed consent and the procedures were in accordance with the Helsinki Declaration. The study was approved by the Ethics Committee of the Saarland Physicians Council (Application 06/08). Aspirates were immediately aliquoted in a volume of 100 μl per well in 96‐well plates and transduced with 40 μl vector (*i.e*. 8 × 10^5^ functional recombinant viral particles, MOI = 10 ± 3) 22, 23. Samples were incubated for up to 21 days with various differentiation medium (Table 1), with careful weekly medium change to induce chondro‐, osteo‐, and adipogenic differentiation 22, 23. Under continuous chondrogenic induction, mostly MSCs in the samples will commit towards the chondrocyte phenotype 23. As we previously noted that the use of these media does not impede the effects of gene transfer *via* rAAV on the differentiation processes *versus* basal medium in aspirates 23, uninduced conditions were not further tested here.

**Table 1:** Inductive media for chondrogenic, adipogenic and osteogenic differentiation

Pathway	Medium
Chondrogenesis	4.5 g/l DMEM high glucose, 100 U/ml penicillin, 100 μl/ml streptomycin, 6.25 μg/ml insulin, 6.25 μg/ml transferrin, 6.25 μg/ml selenious acid, 5.35 μg/ml linoleic acid, 1.25 μg/ml BSA, 1 mM sodium pyruvate, 37.5 μg/ml ascorbate 2‐phosphate, 10^−7^ M dexamethasone and 10 ng/ml TGF‐β3
Osteogenesis	StemPro Osteogenesis Differentiation Kit (Life Technologies, Darmstadt, Germany)
Adipogenesis	StemPro Adipogenesis Differentiation Kit (Life Technologies)

### Transgene expression

Transforming growth factor‐β production was monitored by ELISA at the denoted time‐points by absorbance measurements on a GENios spectrophotometer/fluorometer (Tecan, Crailsheim, Germany) and by immunohistochemistry using a specific TGF‐β antibody, a biotinylated secondary antibody, and diaminobenzidine as a chromogen (ABC method) 28, 31. A control condition with omission of the primary antibody was included to check for secondary immunoglobulins. All sections were examined under light microscopy (Olympus BX45; Olympus, Hamburg, Germany).

### Biochemical analyses

The aspirates were resuspended in a total volume of 100 μl of fresh DMEM and digested with papain (final concentration 75 μg/ml) at 60°C 23. The DNA contents were measured by fluorimetry using Hoechst 22358 and the proteoglycan contents by binding to dimethylmethylene blue dye 23. The type‐II, ‐I and ‐X collagen contents were determined by ELISA 23. Values were normalized to total cellular proteins monitored *via* Pierce Thermo Scientific Protein Assay (Fisher Scientific, Schwerte, Germany).

For the determination of ALP activity, osteogenically induced aspirates were mixed with an equal volume of substrate buffer (4‐nitrophenyl phosphate – pNPP ‐ mixed 1:1 with 4.8% 2‐amino‐2‐methyl‐1‐propanol – 2‐AMP) for measurement of OD^530nm^ 23. Adipogenically induced samples were resuspended in 150 μl of staining solution (three volumes of Oil Red O 0.3% in 2‐propanol and two volumes H_2_O) and incubated for 15 min. at room temperature followed by dissolution in 100% 2‐propanol and by measuring OD^530nm^ 23. All measurements were performed on a GENios spectrophotometer/fluorometer (Tecan).

### Histological and immunohistochemical analyses

Aspirates were collected and fixed in 4% formalin with subsequent dehydration in graded alcohols, paraffin embedding and sectioning at 3 μm. Haematoxylin and eosin staining was performed to evaluate cellularity and toluidine blue and alizarin red staining for the detection of matrix proteoglycans and matrix mineralization respectively 22, 23. The expression of type‐II, ‐I and ‐X collagen and of SOX9 was evaluated by immunohistochemistry using specific primary antibodies, biotinylated secondary antibodies and the ABC method 22, 23. Control conditions were included by omitting the primary antibodies. All sections were examined under light microscopy (Olympus BX45; Olympus).

### Histomorphometry

Transgene expression was monitored by evaluating the percentage of TGF‐β^+^ cells to the total number of cells on immunohistochemical sections 23. Cell proliferation was evaluated by counting the total cells per standardized area on haematoxylin and eosin‐stained sections 23. The intensities of toluidine blue and alizarin red staining and those of type‐II, ‐I and ‐X collagen and SOX9 immunostaining were monitored at magnification ×20 by inverting the pictures to greyscale mode, adapting background DAB signal for comparable range and measuring the mean grey value per total area covered with cells (mm^2^) 23. The data were recorded at three random standardized sites or with 10 serial histological and immunohistochemical sections for each parameter, test and replicate condition using the SIS analySIS program (Olympus) and Adobe Photoshop (Adobe Systems, Unterschleissheim, Germany) and are given as mean intensity of staining or immunostaining per mm^2^ of total cell area 23.

### Real‐time RT‐PCR analyses

TRIzol reagent (Ambion^®^ Life Technologies, Thermo Scientific, Schwerte, Germany) and RNeasy Protect Mini Kit (Qiagen, Hilden, Germany) were used to extract total cellular RNA from all chondrogenically differentiated aspirates on day 21 post‐transduction. The procedure included an on‐column RNase‐free DNase treatment (Qiagen) and extracted RNA was eluted in 30 μl of RNase‐free water followed by reverse transcription using the 1st Strand cDNA Synthesis kit for RT‐PCR (AMV; Roche Applied Science, Mannheim, Germany) with aliquots of 8 μl RNA eluate. The resulting cDNA products (≥2 μl per sample) were finally amplified by real‐time RT‐PCR with Brilliant SYBR Green QPCR Master Mix (Stratagene, Agilent Technologies, Waldbronn, Germany) on an Mx3000P QPCR operator system (Stratagene) under the following conditions: (95°C, 10 min.), amplification by 55 cycles (denaturation at 95°C, 30 sec.; annealing at 55°C, 1 min.; extension at 72°C, 30 sec.), denaturation (95°C, 1 min.) and final incubation (55°C, 30 sec.). Primers for selected gene profiles are listed in Table 2 and applied at a final concentration of 150 nm. Controls consisting of water and non‐reverse‐transcribed mRNA were included and confirmation of the product specificities was done *via* melting curve analysis and agarose gel electrophoresis as previously described 23. The MxPro QPCR Software (Stratagene) was used for measurements of the threshold cycle (Ct) value of each gene of interest and all values were normalized to GAPDH expression using the 2^−ΔΔCt^ method 23.

**Table 2:** Primers used for RT‐PCR

Gene	Primer sequences (5′–3′)
SOX9^a^	ACACACAGCTCACTCGACCTTG GGGAATTCTGGTTGGTCCTCT
COL2A1^a^	GGACTTTTCTCCCCTCTCT GACCCGAAGGTCTTACAGGA
ACAN^a^	GAGATGGAGGGTGAGGTC ACGCTGCCTCGGGCTTC
COL1A1^b^	ACGTCCTGGTGAAGTTGGTC ACCAGGGAAGCCTCTCTCTC
COL10A1^c^	CCCTCTTGTTAGTGCCAACC AGATTCCAGTCCTTGGGTCA
MMP13^c^	AATTTTCACTTTTGGCAATGA CAAATAATTTATGAAAAAGGGATGC
ALP^b^	TGGAGCTTCAGAAGCTCAACACCA ATCTCGTTGTCTGAGTACCAGTCC
RUNX2^b^	GCAGTTCCCAAGCATTTCAT CACTCTGGCTTTGGGAAGAG
GAPDH^d^	GAAGGTGAAGGTCGGAGTC GAAGATGGTGATGGGATTTC

a Chondrogenic markers.

b Osteogenic markers.

c Hypertrophic and terminal differentiation markers.

d Housekeeping gene (control).

SOX9: SRY (sex determining region Y)‐box 9; COL2A1: type‐II collagen α1; ACAN: aggrecan; COL1A1: type‐I collagen α1; COL10A1: type‐X collagen α1; MMP13: matrix metallo‐proteinase 13; ALP: alkaline phosphatase; RUNX2: runt‐related transcription factor 2; GAPDH: glyceraldehyde‐3‐phosphate dehydrogenase.

### Statistical analyses

Each condition was performed in duplicate in two independent experiments for each patient. Data are expressed as mean ± S.D. of separate experiments. The *t*‐test and Mann–Whitney rank‐sum test were used where appropriate. Any *P*‐value of less than 0.05 was considered statistically significant.

## Results

### Effective TGF‐β overexpression *via* rAAV in chondrogenically induced human bone marrow aspirates

**Figure 1:**
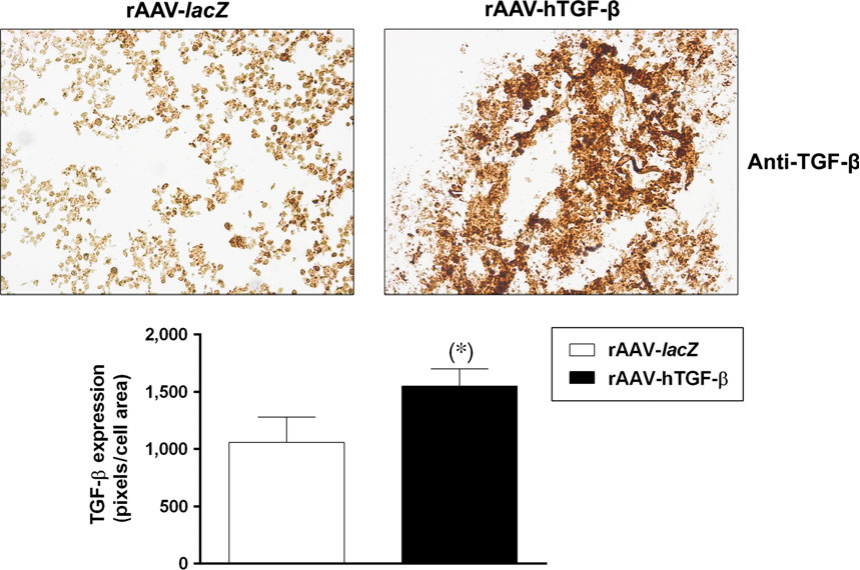
Detection of TGF‐β overexpression *via* rAAV‐mediated gene transfer in chondrogenically induced human bone marrow aspirates. The aspirates were transduced with rAAV‐*lacZ* or rAAV‐hTGF‐β (40 μl each vector) and kept in chondrogenic medium (Table 1) for 21 days. The samples were processed to monitor TGF‐β production by immunohistochemical analysis (magnification ×20; representative data) with corresponding histomorphometric assessments and by estimating the transduction efficiencies as described in the Materials and methods. *Statistically significant compared with rAAV‐*lacZ*.

Human bone marrow aspirates were first transduced with the candidate rAAV‐hTGF‐β vector *versus* control (rAAV‐*lacZ*) condition and cultivated for up to 21 days to test the ability of rAAV to promote the overexpression of the candidate TGF‐β factor in conditions of chondrogenic induction. Transgene expression analyses revealed significantly higher immunoreactivity to TGF‐β in the aspirates transduced with rAAV‐hTGF‐β compared with rAAV‐*lacZ* (1553.2 ± 147.0 and 1056.9 ± 221.1 pixels/cell area after 21 days in the TGF‐β‐ *versus lacZ*‐treated samples, respectively, *i.e*. a 1.5‐fold difference, *P* = 0.002) (Fig. 1). These results were supported by an estimation of the TGF‐β production levels, revealing significantly higher values on days 7 and 21 (up to 2.9‐fold difference, *P* ≤ 0.021) (Table 3).

**Table 3:** Production of TGF‐β in chondrogenically induced human bone marrow aspirates *via* rAAV‐mediated gene transfer

Days post‐transduction	rAAV‐*lacZ*	rAAV‐hTGF‐β
7	178.5 (10.0)	511.1 (116.9)^a^
14	49.4 (7.1)	112.5 (56.3)
21	5.8 (3.1)	16.1 (5.9)^a^

a Statistically significant compared with rAAV‐*lacZ*.

Values are expressed as means pg/24 hrs/mg total proteins (S.D.).

### Proliferative and chondrogenic effects of rAAV‐mediated overexpression of TGF‐β in chondrogenically induced human bone marrow aspirates

The aspirates were next transduced with rAAV‐hTGF‐β compared with rAAV‐*lacZ* and induced towards chondrogenesis to evaluate the effects of TGF‐β overexpression upon the proliferative activities and potential for chondrogenic differentiation in the samples. As we previously reported a lack of deleterious effects of rAAV gene transfer upon the potency of bone marrow aspirates 22, we did not further include a condition without vector treatment in this study.

**Figure 2:**
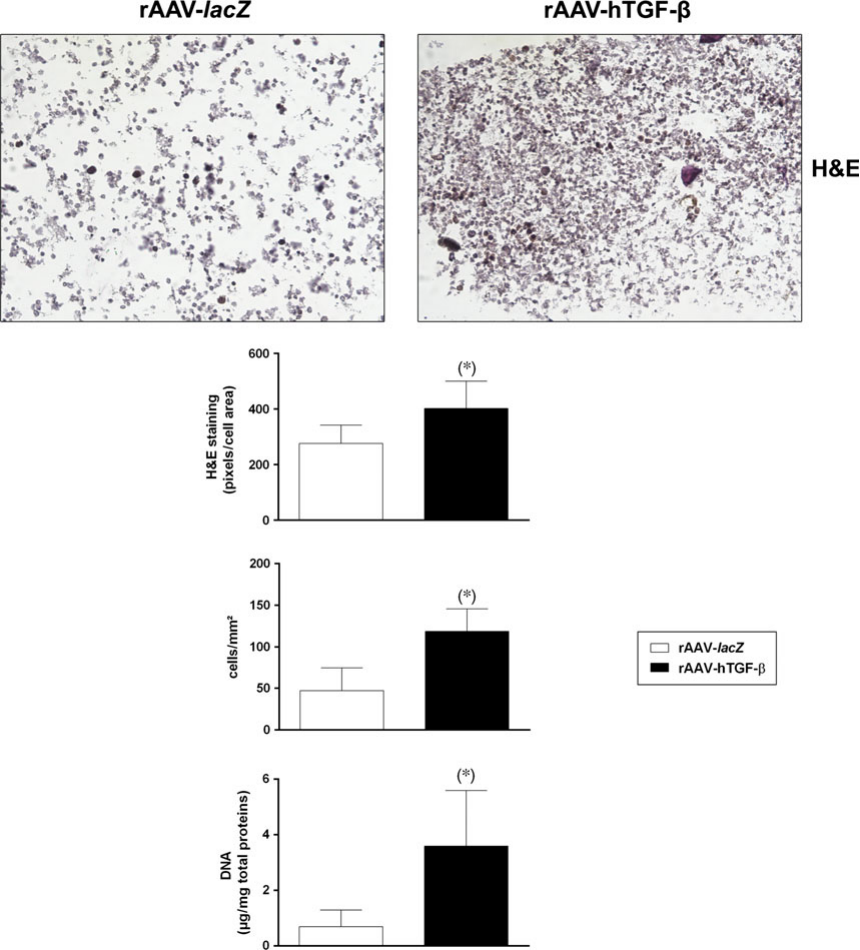
Effects of rAAV‐mediated TGF‐β overexpression upon the proliferative activities in chondrogenically induced human bone marrow aspirates. The aspirates were transduced with rAAV‐*lacZ* or rAAV‐hTGF‐β as described in Figure 1 and kept in chondrogenic medium for 21 days. The samples were processed to evaluate the cell densities on haematoxylin and eosin‐stained histological sections (magnification ×20; representative data) and to monitor the DNA contents by Hoechst 22358 assay as described in the Materials and methods. *Statistically significant compared with rAAV‐*lacZ*.

A quantitative analysis by haematoxylin and eosin staining revealed significantly higher cell densities in the presence of rAAV‐hTGF‐β compared with rAAV‐*lacZ* (402.0 ± 98.9 and 276.0 ± 66.2 pixels/cell area and 118.8 ± 27.1 and 47.2 ± 27.2 cells/mm^2^ after 21 days in the TGF‐β‐ *versus lacZ*‐treated samples, respectively, *i.e*. an up to 2.5‐fold difference, always *P* ≤ 0.004) (Fig. 2). These results were further confirmed *via* a biochemical assay to monitor the DNA contents in the aspirates, revealing significantly higher values with rAAV‐hTGF‐β compared with rAAV‐*lacZ* (3.6 ± 2.0 and 0.7 ± 0.6 μg/mg total proteins after 21 days in the TGF‐β‐ *versus lacZ*‐treated samples, respectively, *i.e*. a 5.1‐fold difference, *P* = 0.018) (Fig. 2).

**Figure 3:**
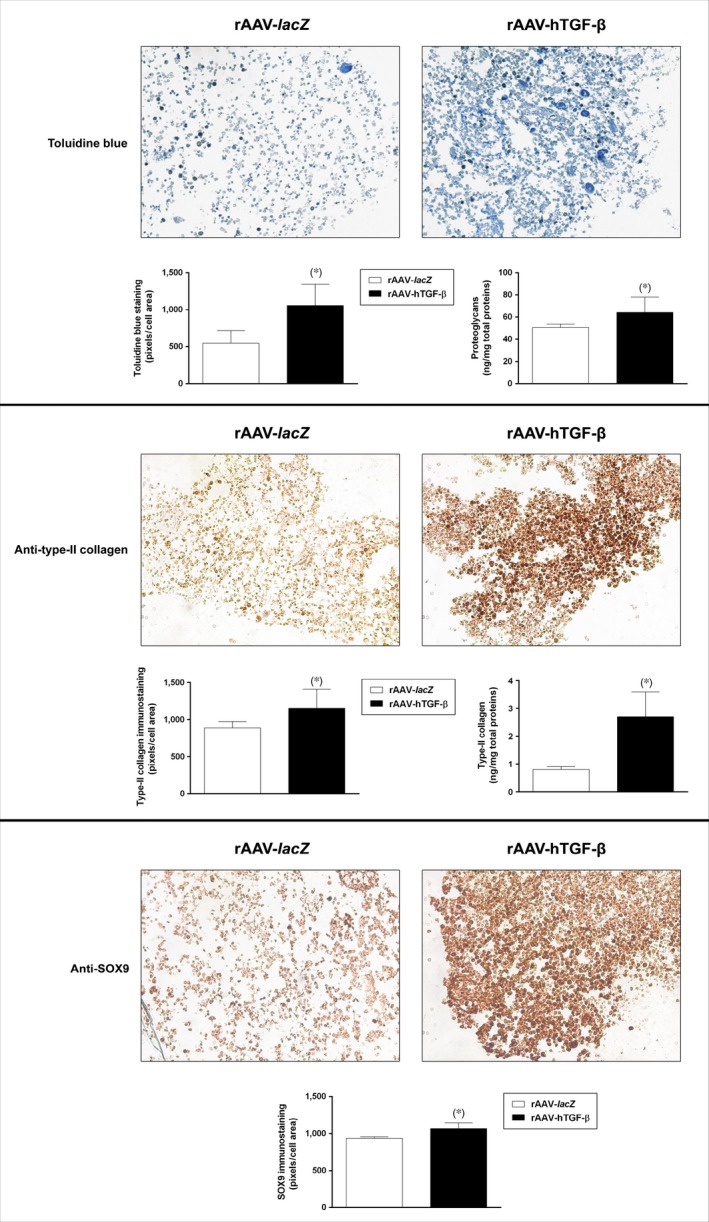
Detection of cartilage‐specific components in chondrogenically induced human bone marrow aspirates overexpressing TGF‐β. The aspirates were transduced with rAAV‐*lacZ* or rAAV‐hTGF‐β as described in the Figures 1 and 2 and kept in chondrogenic medium for 21 days. The samples were processed to monitor the deposition of proteoglycans and of type‐II collagen (toluidine blue staining and immunodetection, respectively) and the expression of SOX9 (immunodetection) (all at magnification ×20; representative data), including histomorphometric analyses and an estimation of the proteoglycan and type‐II collagen contents as described in the Materials and methods. *Statistically significant compared with rAAV‐*lacZ*.

To monitor the effects of TGF‐β overexpression upon the chondrogenic events in the aspirates, the samples were further processed for the cartilage‐specific proteoglycans, type‐II collagen, and SOX9 markers by histological and immunohistochemical analyses. Significantly increased intensities were detected in the aspirates transduced with rAAV‐hTGF‐β compared with rAAV‐*lacZ* for toluidine blue staining (1059.4 ± 284.1 and 545.1 ± 173.7 pixels/cell area after 21 days in the TGF‐β‐ *versus lacZ*‐treated samples, respectively, *i.e*. a 1.9‐fold difference, *P* = 0.005), anti‐type‐II collagen immunostaining (1151.5 ± 260.8 and 888.9 ± 85.8 pixels/cell area after 21 days in the TGF‐β‐ *versus lacZ*‐treated samples, respectively, *i.e*. a 1.3‐fold difference, *P* = 0.037), and anti‐SOX9 immunostaining (1069.8 ± 76.8 and 938.9 ± 20.9 pixels/cell area after 21 days in the TGF‐β‐ *versus lacZ*‐treated samples, respectively, *i.e*. a 1.2‐fold difference, *P* = 0.028) (Fig. 3). A biochemical analysis of the proteoglycan and type‐II collagen contents confirmed these results, showing higher values in the presence of rAAV‐hTGF‐β (64.3 ± 13.8 and 50.8 ± 2.9 ng proteoglycans/mg total proteins and 2.7 ± 0.9 and 0.8 ± 0.1 ng type‐II collagen/mg total proteins after 21 days in the TGF‐β‐ *versus lacZ*‐treated samples, respectively, *i.e*. an up to 3.4‐fold difference, always *P* ≤ 0.047) (Fig. 3). In addition, an analysis of the gene expression profiles in the aspirates by real‐time RT‐PCR revealed increases in the expression of ACAN (1.6‐fold), COL2A1 (1.4‐fold), and SOX9 (6.8‐fold) after 21 days in the aspirates transduced with rAAV‐hTGF‐β compared with rAAV‐*lacZ* (always *P* ≤ 0.028) (Fig. 5).

**Figure 4:**
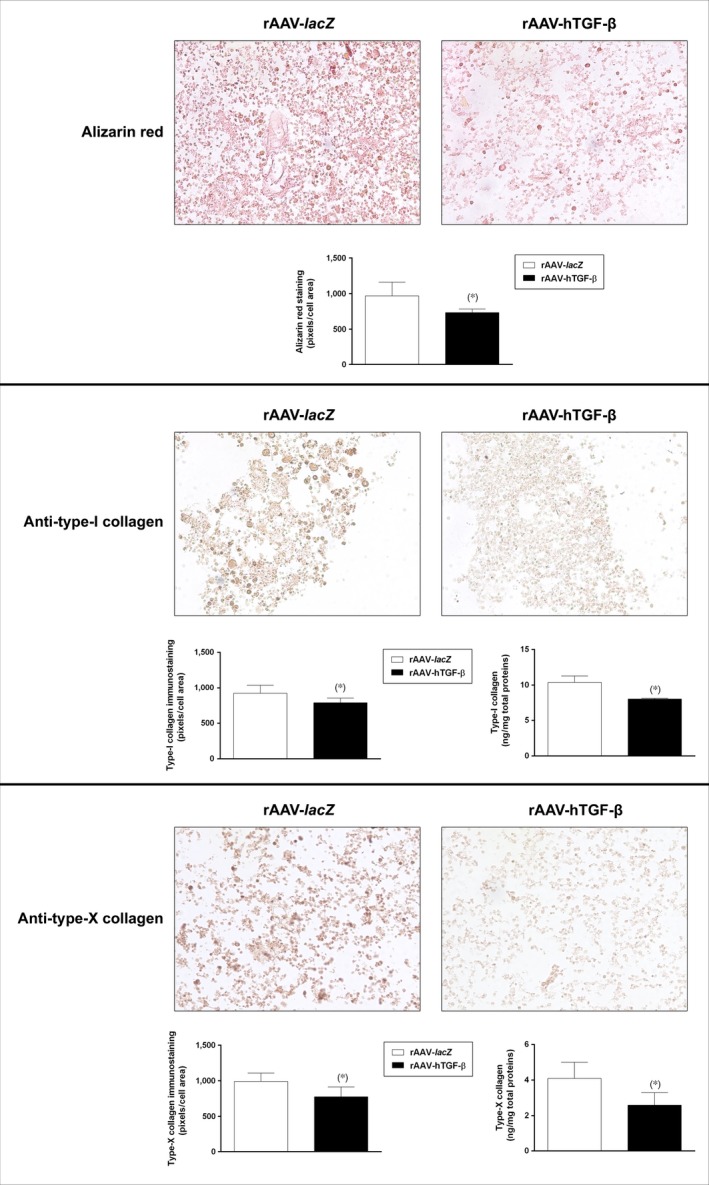
Detection of hypertrophy and terminal differentiation in chondrogenically induced human bone marrow aspirates overexpressing TGF‐β. The aspirates were transduced with rAAV‐*lacZ* or rAAV‐hTGF‐β as described in the Figures 1, 2, 3 and kept in chondrogenic medium for 21 days. The samples were processed to monitor matrix mineralization (alizarin red staining) and the expression of type‐I and type‐X collagen (immunodetection) (all at magnification ×20; representative data), including histomorphometric analyses and an estimation of the type‐I and ‐X collagen contents as described in the Materials and methods. *Statistically significant compared with rAAV‐*lacZ*.

**Figure 5:**
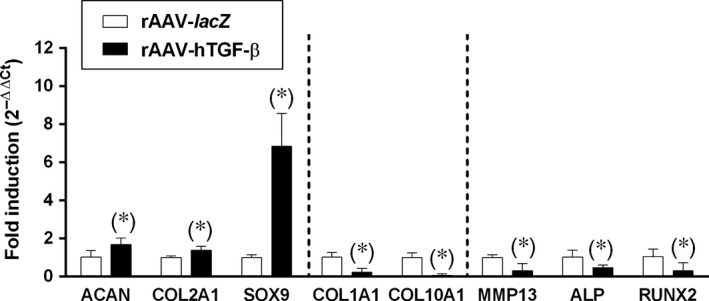
Gene expression analyses by real‐time RT‐PCR in chondrogenicallyinduced human bone marrow aspirates overexpressing TGF‐β. The aspirates were transduced with rAAV‐*lacZ* or rAAV‐hTGF‐β as described in the Figures 1, 2, 3, 4 and kept in chondrogenic medium for 21 days. The samples were processed to monitor the expression profiles of aggrecan (ACAN), type‐II collagen (COL2A1), the transcription factor SOX9, type‐I collagen (COL1A1), type‐X collagen (COL10A1), matrix metallo‐proteinase 13 (MMP13), alkaline phosphatase (ALP), and the transcription factor RUNX2, with GAPDH serving as a housekeeping gene and internal control for normalization. Ct values were generated for each target gene and fold inductions (relative to rAAV‐*lacZ*‐treated aspirates) were measured by using the 2^−ΔΔCt^ method as described in the Materials and methods. *Statistically significant compared with rAAV‐*lacZ*.

Hypertrophic/terminal differentiation was also monitored in the aspirates to evaluate the impact of TGF‐β overexpression in the samples. An immunohistochemical analysis revealed significantly lower intensities in the aspirates transduced with rAAV‐hTGF‐β compared with rAAV‐*lacZ* for alizarin red staining (734.9 ± 48.0 and 968.7 ± 192.6 pixels/cell area after 21 days in the TGF‐β‐ *versus lacZ*‐treated samples, respectively, *i.e*. a 1.3‐fold difference, *P* = 0.016), type‐I collagen immunostaining (789.5 ± 64.7 and 921.2 ± 117.9 pixels/cell area after 21 days in the TGF‐β‐ *versus lacZ*‐treated samples, respectively, *i.e*. a 1.2‐fold difference, *P* = 0.043), and type‐X collagen immunostaining (777.4 ± 136.1 and 992.3 ± 116.3 pixels/cell area after 21 days in the TGF‐β‐ *versus lacZ*‐treated samples, respectively, *i.e*. a 1.3‐fold difference, *P* = 0.001) (Fig. 4). A biochemical analysis performed to estimate the type‐I and ‐X collagen contents supported these findings, showing lower values in the presence of rAAV‐hTGF‐β (8.0 ± 0.1 and 10.4 ± 0.9 ng type‐I collagen/mg total proteins and 2.6 ± 0.7 and 4.1 ± 0.9 ng type‐X collagen/mg total proteins after 21 days in the TGF‐β‐ *versus lacZ*‐treated samples, respectively, up to 1.6‐fold difference, always *P* ≤ 0.041) (Fig. 4). Also, an analysis of the gene expression profiles in the aspirates monitored by real‐time RT‐PCR revealed decreases in the expression of type‐I (4.3‐fold) and type‐X collagen (13.1‐fold) after 21 days in the aspirates transduced with rAAV‐hTGF‐β compared with rAAV‐*lacZ* (always *P* ≤ 0.003) (Fig. 5). Such profiles were accompanied by a reduced expression of markers of terminal differentiation and osteogenesis with rAAV‐hTGF‐β (3.2‐, 2.2‐, and 3.5‐fold decreased MMP13, ALP, and RUNX2 expression after 21 days, respectively), although statistical significance was not reached in the conditions applied here (always *P* ≤ 0.210) (Fig. 5).

### Effects of rAAV‐mediated overexpression of TGF‐β in osteogenically and adipogenically induced human bone marrow aspirates

**Figure 6:**
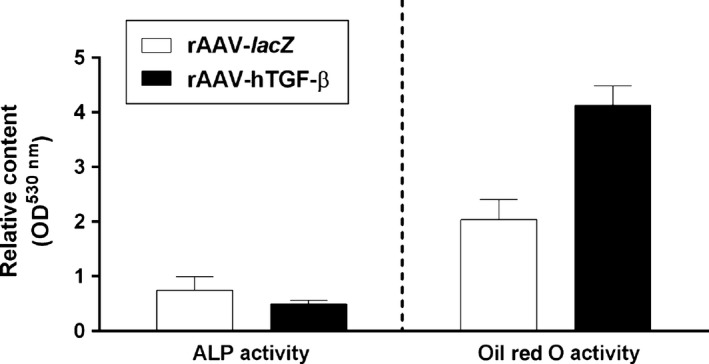
Detection of osteogenic and adipogenic differentiation in osteogenically and adipogenically induced human bone marrow aspirates overexpressing TGF‐β. The aspirates were transduced with rAAV‐*lacZ* or rAAV‐hTGF‐β as described in the Figures 1, 2, 3, 4, 5 and kept either in osteogenic or adipogenic medium (Table 1) for 21 days. The samples were processed to monitor the ALP activities and evaluate Oil Red O staining, respectively, as described in Materials and methods.

The aspirates were also transduced with rAAV‐hTGF‐β compared with rAAV‐*lacZ* and induced towards osteogenesis and adipogenesis to evaluate the influence of TGF‐β overexpression on the potential for osteo‐/adipogenic differentiation in the samples. While a trend towards enhanced adipogenic differentiation (Oil Red O staining) was seen in the presence of rAAV‐hTGF‐β compared with rAAV‐*lacZ* (4.13 ± 0.36 and 2.04 ± 0.37 OD^530nm^ after 21 days in the TGF‐β‐ *versus lacZ*‐treated samples, respectively, *i.e*. a twofold difference), osteogenic differentiation (ALP activity) was attenuated with TGF‐β (0.49 ± 0.07 and 0.75 ± 0.25 OD^530nm^ after 21 days in the TGF‐β‐ *versus lacZ*‐treated samples, respectively, *i.e*. a 1.5‐fold difference), although statistical significance was not reached in the conditions applied here (*P* = 0.076 and *P* = 0.234 respectively) (Fig. 6).

## Discussion

Administration of genetically modified marrow concentrates to site of cartilage lesions *via* transfer of chondrogenic and/or chondroreparative factors using the clinically adapted rAAV vectors may provide convenient, single‐step therapeutic approaches to enhance cartilage repair compared with the more complex and invasive implantation of isolated progenitor cells 6. In this study, we examined the ability of rAAV to deliver a functional TGF‐β gene cassette to human marrow concentrates in the light of the pleiotropic, chondrogenic properties of this growth factor 24, 25, 26, 27 and with our previous findings showing that such a strategy was capable of stimulating such activities in isolated human MSCs 28.

We first observed that transduction with rAAV led to higher levels of TGF‐β expression in the hTGF‐β‐treated concentrates compared with control (*lacZ*) treatment over the period of evaluation (21 days), in good agreement with our previous results in hMSCs 28. The concentrations achieved *via* rAAV‐hTGF‐β gene transfer over time were in the range of those noted in hMSCs (~16.1 and ~24.1 pg rhTGF‐β/mg total proteins on day 21 in the aspirates and in isolated cells respectively) 28. Our findings further indicate that overexpression of TGF‐β *via* rAAV led to increased levels of proliferative, biosynthetic and chondrogenic activities in the aspirates over time relative to control treatment, possibly because of the prolonged production of the growth factor permitted by stable rAAV gene transfer 32 and concordant with the effects of TGF‐β when applied in a recombinant form 24, 25, 26, 27 or used by us to target isolated hMSCs 28. Of note, the levels of proliferation in the TGF‐β‐treated aspirates were higher than those noted with hMSCs (~3.6 μg *versus* ~26.4 ng DNA/mg total proteins, respectively, *i.e*. a 136‐fold difference) 28, possibly because of the presence of other mitogenic factors in the aspirates and/or to effects from other marrow cell populations modified early on by rAAV while later on, under continuous chondrogenic activation mostly chondrogenically induced, rAAV‐modified MSCs may control this process (paracrine *versus* autocrine effects). We thus anticipate that their depletion may prevent the effects of TGF‐β in conditions of permanent chondrogenic activation over the current extended period of evaluation. Still, work would be needed to confirm this hypothesis by modifying aspirates devoid of the MSC subpopulation. In contrast, the levels of matrix synthesis were lower in the aspirates compared with those observed in isolated hMSCs (~2.7 *versus* ~9.5 ng type‐II collagen/mg total proteins on day 21, respectively, *i.e*. a 3.5‐fold difference; ~64.3 ng *versus* ~378.5 μg proteoglycans/mg total proteins, respectively, *i.e*. a 5.8 × 10^3^‐fold difference) 28, possibly because of a lower state of activation of chondroprogenitor cells in the aspirates compared with that acquired during cell expansion, to adverse effects from distinct, residual cells, and/or to prevalent mitogenic *versus* anabolic effects of TGF‐β in aspirates as noted in chondrocytes 33. It cannot be excluded that the levels of matrix synthesis may still increase upon TGF‐β treatment beyond the time‐point selected here according to the relevant literature 19, 20, 23. Such effects are most likely mediated by binding of TGF‐β to its receptor (TβRII‐TβRI complex with R‐Smad cascade) and work is ongoing to test this hypothesis by performing receptor blocking studies 34. Finally, we report that in the conditions tested here, application of the candidate rAAV‐hTGF‐β vector delayed hypertrophic and osteogenic differentiation in the aspirates, in association with reduced levels of MMP13 (marker of terminal differentiation), ALP (osteogenic marker) and RUNX2 (transcription factor controlling the osteoblastic expression of COL1, COL10 and MMP13). This is in striking contrast with our previous findings in isolated hMSCs, where the same vector stimulated instead these processes compared with control treatment 28. Again, this might be the result of effects from other, beneficial factors present in the aspirates and/or of specific control pathways possibly activated in the presence of other marrow cell populations 35.

In conclusion, the results of this study show the value of providing therapeutic rAAV vectors to human bone marrow concentrates to enhance the chondrogenic differentiation processes for implantation in sites of cartilage injury. Work is ongoing to support the practicability of the approach by applying modified aspirates to focal defects 6, 19, 20, 21, 36, 37. Also important, the cell subpopulations promoting the chondrogenic events in the aspirates are currently being characterized 7, 8, 38 to address the cell heterogeneity of the aspirates. Also, it remains to be seen whether gene transfer of TGF‐β will be sufficient to achieve full repair in the lesions, and co‐application of other candidates might be necessary, like for instance the cartilage oligomeric matrix protein, BMPs, IGF‐I, basic fibroblast growth factor‐2, SOX transcription factors, zinc‐finger protein 145 (ZNF145), Indian hedgehog (Ihh) or human telomerase (hTERT) 22, 23, 39, 40, 41, 42, 43, 44, 45, 46, 47. Combined gene transfer will be possible using rAAV as transduction with this class of vectors does not lead to gene transfer interference 48. Overall, our results show the benefits of a direct modification of marrow aspirates *via* rAAV for the future treatment of cartilage defects.

## Conflicts of interest

The authors confirm that there are no conflicts of interest.
